# Impact of pri-let-7a-1 rs10739971 for Gastric Cancer Predisposition in an Amazon Region

**DOI:** 10.3390/genes14020453

**Published:** 2023-02-09

**Authors:** Roberta Borges Andrade, Amanda de Nazaré Cohen-Paes, Diana Feio da Veiga Borges Leal, Karla Beatriz Cardias Cereja Pantoja, Laura Patrícia Albarello Gellen, Darlen Cardoso de Carvalho, Tatiane Piedade de Souza, Marianne Rodrigues Fernandes, Paulo Pimentel de Assumpcão, Rommel Mario Rodríguez Burbano, Sidney Emanuel Batista dos Santos, Ney Pereira Carneiro dos Santos

**Affiliations:** 1 Oncology Research Center, Federal University of Pará, Belém 66073-000, Pará, Brazil; 2 Ophir Loyola Hospital, Molecular Biology Laboratory, Belém 66063-240, Pará, Brazil

**Keywords:** gastric cancer, cancer predisposition, Amazon

## Abstract

Gastric cancer (GC) is the fifth most common type of cancer and the fourth leading cause of cancer death. In Brazil, GC has a high incidence and mortality rates, and it is highly variable by region. The Amazon region has significant rising rates among all Brazil regions. Only very few studies have evaluated the association between genetic variants and the risk of gastric cancer in the Brazilian Amazon population. Therefore, this study aimed to investigate associations between single nucleotide polymorphisms of miRNA processing genes and the risk for GC in this population. Potentially functional single nucleotide polymorphisms from miRNA processing genes were genotyped in 159 cases and 193 healthy controls by QuantStudio Real Time PCR. According to our findings, the genotype GG of the variant rs10739971 presents a lower risk to the development of GC in comparison to the remaining genotypes (*p* = 0.000016; OR = 0.055; 95% CI = 0.015–0.206). This is the first study to report the association of pri-let-7a-1 rs10739971 with GC in the Brazilian Amazon population, which is a highly mixed population with a unique genetic constitution that is different from other populations that are studied in the vast majority of scientific research.

## 1. Introduction

Worldwide, more than 18 million new cases and approximately 10 million cancer deaths occurred in 2020 [1]. Gastric cancer (GC) is the fifth most common cancer and the fourth leading cause of cancer death [1,2]. GC is known to be a complex and heterogeneous disease with genetic factors, such as the alteration of important genes (e.g., *TP53* and *CDH1*); environmental factors, such as infection by *Helicobacter pylori*, bad eating habits, alcohol consumption and smoking; and epigenetic factors, which involve DNA methylation, histone modification and small and long non-coding RNAs [3]. In Brazil, this neoplasm has a high incidence and mortality rates, especially in the northern region [4].

GC is frequently not diagnosed until an advanced stage. Early detection and monitoring of tumors are prerequisites for reducing disease epidemiology and mortality rates [5,6,7]. There is an urgent need to discover biomarkers for the non-invasive early detection of gastric cancer patients in several world populations. Many investigations are searching for accurate biomarkers to detect gastric cancer [4,8,9,10,11].

Recent research to identify biomarkers in gastric cancer has resulted in the discovery of a wide variety of cancer-related molecules, including microRNAs (miRNAs). They play roles in several physiologic processes and are mostly located in cancer-associated genomic regions. Dysregulation of miRNA has been reported in tumor initiation, progression, invasion, and metastasis [12,13,14], and it is involved in gastric cancer, acting as a tumor suppressor or oncogenic microRNAs [7,15,16,17].

Studies reinforce the relevant role of SNPs in genes of miRNA with associations to different types of cancer [18,19,20], including gastric cancer [20]. Specifically, polymorphisms that alter microRNAs in their binding sites might rupture the regulation of important biological pathways implicated in tumorigenesis and tumor progression [21]. Despite this, there are still few studies like this one in the literature, and even fewer studies on these polymorphisms in populations with a high rate of admixture, such as the one in the Brazilian Amazon.

An early gastric cancer diagnosis is essential to managing patients. The current gold standard method to detect GC is an upper endoscopy and tissue biopsy, with low sensitivity and specificity values. Therefore, it is important to discover new noninvasive biomarkers able to diagnose the risk of gastric cancer and to significantly improve the overall outcome [5,6,7]. Thus, the present study investigated the role of 26 variants in genes of microRNAs and genes of miRNA synthesis in the susceptibility to gastric cancer in an admixed population from the Brazilian Amazon, where the incidence and mortality rates of this type of cancer are high.

## 2. Materials and Methods

### 2.1. Study Populations

This study was approved by the Research Ethics Committee of the Center of Research in Oncology of the Federal University of Pará (CAAE nº 11433019.5.0000.5634). The participants of the research were chosen based on the structure of a case-control study. The case group was composed of 159 patients diagnosed with gastric adenocarcinoma at the University Hospital João de Barros Barreto (HUJBB) and at the Hospital Ophir Loyola (HOL). The control group consisted of 193 unrelated and cancer-free individuals from the same geographic area as the case group.

All participants are from the northern region of Brazil, which presents a great ethnic admixture between Native Americans, Europeans and Africans, whose individual genetic contributions varied substantially between 5 and 47% for African genes, 16 and 86% for European genes and 9 and 68% for Native American genes (as described by Santos et al.) [22]. 

### 2.2. Extraction and Quantification of the DNA

DNA was extracted from peripheral blood using the commercial BiopurKit Mini Spin Plus Extraction Kit—250 (Biopur, Pinhais, PR, Brazil), according to the manufacturer’s instructions. Samples were quantified on the NanoDrop 1000 Spectrophotometer (Thermo Fisher Scientific, Waltham, MA, USA).

### 2.3. Genotyping of Polymorphisms

Genotyping of polymorphisms was performed by QuantStudioTM–TaqMan^®^ OpenArrayTM (Thermo, Carlsbad, CA, USA) based on real-time polymerase chain reaction (RT-PCR). The reactions were performed on a plate, where each well contained 2 μL of DNA from each patient and 2 μL of TaqMan^®^ OpenArray^®^ Genotyping Master Mix (Thermo, Carlsbad, CA, USA), for subsequent pipetting on the customized OpenArrayTM chip. Then, the amplification of the DNA fragments where the SNPs would be located was performed in QuantStudio (Thermo Fisher Scientific). Results were analyzed using TaqMan^®^ v1.2 Genotyper software (Thermo Fisher Scientific).

We initially selected 26 polymorphisms that were previously related to cancer and/or gastric cancer. To ensure an adequate level of accuracy, the polymorphisms were selected for further analysis according to three criteria: (i) MAF ≥ 1%; (ii) genotyping rate ≥ 80%; and (iii) if it was in Hardy–Weinberg equilibrium (HWE). The HWE was performed using the Arlequin software v.3.5.1.2 (Institute of Ecology and Evolution, University of Bern, Switzerland). Once these criteria were applied, only 16 polymorphisms were selected for the association analysis (Table S1).

### 2.4. Statistical Analysis

Statistical analyses were performed with R package v.3.4.3 (R foundation for statistical computing, http://www.r-project.org (accessed on 15 October 2022), considering a level of significance of *p* ≤ 0.05. The effect of the variants on the risk of gastric cancer was evaluated using multiple logistic regression, adjusted for sex and age. For each variant, recessive, dominant and log-additive genetic models were evaluated, and the best genetic model was selected using the Akaike information criterion (AIC), available in the package “SNPassoc” version 1.9-2 (https://cran.rproject.org/web/packages/SNPassoc/index.html (accessed on 15 October 2022) for R software. The significance of tests was adjusted for multiple comparison by Bonferroni correction (adjusted *p* Value = 0.003125).

## 3. Results

Individuals from the case group had significantly lower ages and a predominance of males when compared to the control group (Table 1). Thus, these potentially confounding variables were controlled in a logistic regression analysis.

Through the Bonferroni correction, the *p*-values were corrected for the 16 variants included in the analyses, which was the number of tests performed (Plimit = 0.003125). Afterwards, only the variant pri-let-7a-1 (rs10739971) remained statistically significant. Data referring to the other variants are described in Table 2.

Regarding the variant rs10739971, the genotype AA presented a lower risk to the development of GC in comparison to the remaining genotypes (*p* = 0.000016; OR = 0.055; 95% CI = 0.015–0.206). These results are described in Table 2.

## 4. Discussion

MicroRNAs are molecules that act in gene regulation and play an important role in the functioning of many biological pathways, being widely recognized as regulators of approximately 30% of genes in humans [6,23]. In the specialized literature, most studies on miRNA and diseases involve methods of gene expression analysis, however, methodologies that directly detect miRNA molecules have a low sensitivity due to the extremely short sequences of miRs and relatively low copy numbers [24]. According to recent investigations, in situ hybridization, one of the most used methods, has a low yield and a limited sensitivity and specificity [25,26]. All this points to an advantage for the methodologies of identification of specific variants of genes of the miRNA machinery [27].

Variants in miRNAs can act directly on disease predisposition, which can affect miRNA maturation or function, or they can act indirectly [28]. Thus, genetic alterations in microRNA genes and involvement in their biosynthesis have been associated with a modulation in the risk of developing several types of cancer, including gastric carcinogenesis [6,29,30,31,32]. In the present study, we report for the first time the association of SNP pri-let-7a-1 rs10739971 to gastric cancer in the population of the Brazilian Amazon region. It is known that genetic variants that are in miRNA genes, including pri regions, would have the chance of affecting several biological pathways and influencing the incidence of diseases. Therefore, polymorphisms in pri regions might be used as genetic markers to predict the risk of cancer development [33].

Let-7a was the first miRNA to be discovered, and today it is one of the most investigated miRNAs, playing a critical role in cell proliferation and differentiation [29,30]. Several studies show that let-7 miRNA participates in the tumorigenesis and metastasis of different types of cancers [34,35,36]. This miRNA targets oncogenes and important genes for the initiation and progression of the tumor, including Myc, RAS, E2F1, E2F5, LIN28, ARID3B, PBX3, HMGA2 and long non-coding RNA H19 [37]. The silencing of these genes causes let-7-mediated functional tumor suppressor effects. An example is PBX3, which is an oncogene that induces epithelial to mesenchymal transition and promotes invasion and metastasis of GC. Therefore, let-7 might act by repressing the function of factors that may be recruited in oncogenesis [38]. Corroborating our study, the GG genotype or the G allele has been associated with an increased risk of GC development or even with a poor prognosis of the disease in the Chinese population; this study also showed that the A allele is associated with a higher survival rate of patients with GC and that it is beneficial to the maturation of miRNA let-7a, indicating that this polymorphism acts as a tumor suppressor by increasing the expression of the mature miRNA [28], which in turn suggests that this SNP may be important to carcinogenesis and to GC survival. On the other hand, the study by Xu et al. [39] did not observe a relation between cancer and polymorphisms in let-7.

It is important to highlight that the Brazilian Amazon population is one of the largest mixed populations in the world, being formed mostly by the contribution of three ancestral populations: European, African and Amerindian. Although miRNA let-7a is extensively studied, most studies were conducted in North American, European or Asiatic populations, and their findings cannot be applied to our admixed population.

Besides the importance of our results, we suggest that further case-control studies with a higher sample number should be performed to validate our findings. We also suggest that novel studies should be conducted with other continental populations, and that the investigation of the expression of miRNA pri-let-7a-1 might be important in determining the impact of this variant.

In summary, the genes investigated here are involved in important biological processes and might play a relevant role in the development of gastric cancer. The results of our work are unique in the global scenario, since our study population is highly admixed with a genetic constitution differentiated from ethnically homogeneous populations that compose other studies. Lastly, from a methodological point of view, the usage of SNPs as biomarkers is advantageous when compared to mRNA expression, since germline genetics can be easily investigated from peripheral blood with less laborious and less costly techniques.

## 5. Conclusions

Our results demonstrated that the variant rs10739971 proved to be important to modulating genetic susceptibility to GC in a population in the Brazilian Amazon, a highly mixed population with a unique genetic constitution that is different from the other populations that are studied in the vast majority of scientific research.

## Figures and Tables

**Table 1:** Demographic variables for patients with gastric cancer and the control group.

Variables	Case	Control	*p*-Value
	159	193	
Age, years *	57.65 ± 15.11	65.97 ± 16.02	<0.001 ^b^
Sex (Female/Male)	67/107	126/52	<0.001 ^a^

^a^ Chi-square; ^b^ Significance determined by Student’s t-test; * Values are expressed as mean (±SD = standard deviation).

**Table 2:** Distribution of the alleles and genotypes of the polymorphisms investigated in the present study in patients with gastric cancer in comparison with the control group.

Genotype	Case (%)	Control (%)	*p*-Value ^a^	OR (95%CI) *
pri-let-7a-1 (rs10739971)	147	83	0.000016	AA vs. Others ^b^
GG	75 (51.0)	32 (38.6)		0.055 (0.015–0.206)
GA	67 (45.6)	33 (39.8)		
AA	5 (3.4)	18 (21.7)		
Allele G	0.7	0.6		
Allele A	0.3	0.4		
MIR323B (rs56103835)	179	142	0.108	CC vs. Others ^b^
TT	85 (47.5)	81 (57)		3.13 (1.1–8.85)
TC	76 (42.5)	54 (38)		
CC	18 (10)	7 (5)		
Allele T	0.7	0.7		
Allele C	0.3	0.3		
MIR2053 (rs10505168)	171	122	0.071	TC vs. Others ^b^
TT	61 (35.6)	56 (46)		2.16 (1.27–3.67)
TC	95 (55.6)	47 (38.5)		
CC	15 (8.8)	19 (15.5)		
Allele T	0.6	0.7		
Allele C	0.4	0.3		
MIR300 (rs12894467)	180	175	0.06470	TT vs. Others ^b^
TT	59 (32.7)	75 (42.8)		1.57 (0.97–2.55)
TC	92 (51.1)	80 (45.7)		
CC	29 (16.1)	20 (11.4)		
Allele T	0.6	0.7		
Allele C	0.4	0.3		
MIR146A (rs2910164)	181	175	0.4131407	GC vs. Others ^b^
GG	67 (37)	68 (42.5)		1.22 (0.76–1.97)
GC	101 (55.8)	74 (46.2)		
CC	13 (7.2)	18 (11.2)		
Allele G	0.6	0.7		
Allele C	0.4	0.3		
MIR196A2 (rs11614913)	172	145	0.09714	TT vs. Others ^b^
CC	100 (58.1)	65 (44.8)		0.44 (0.16–1.19)
CT	63 (36.7)	65 (44.8)		
TT	9 (5.2)	15 (10)		
Allele C	0.8	0.7		
Allele T	0.2	0.3		
AGO1 (rs636832)	173	150	0.5449	AA vs. Others ^b^
GG	72 (41.6)	69 (46)		1.28 (0.58–2.83)
GA	79 (45.7)	67 (44.6)		
AA	22 (12.7)	14 (9.3)		
Allele G	0.6	0.7		
Allele A	0.4	0.3		
MIR608 (rs4919510)	181	161	0.3178	CC vs. Others ^b^
CC	107 (59.2)	85 (52.8)		0.78 (0.48–1.27)
CG	58 (32)	58 (36)		
GG	16 (8.8)	18 (11.2)		
Allele C	0.8	0.7		
Allele G	0.2	0.3		
MIR499 (rs3746444)	181	167	0.06739	GG vs. Others ^b^
AA	148 (81.7)	126 (75)		0.0 (0.0–0.0)
AG	33 (18.3)	36 (22)		
GG	0	5 (3)		
Allele A	0.9	0.9		
Allele G	0.1	0.1		
MIR-200C (rs12904)	155	156	0.4670	GG vs. Others ^b^
GG	35 (22.5)	46 (29.5)		1.24 (0.7–2.19)
GA	79 (51)	72 (46.1)		
AA	41 (26.5)	38 (24.4)		
Allele G	0.5	0.5		
Allele A	0.5	0.5		
MIR4513 (rs2168518)	182	163	0.3813	GG vs. Others ^b^
GG	75 (41.2)	80 (49.1)		1.24 (0.77–2)
GA	85 (46.7)	61 (37.4)		
AA	22 (12.1)	22 (13.5)		
Allele G	0.6	0.7		
Allele A	0.4	0.3		
MIR423 (rs6505162)	157	124	0.05312	AC vs. Others ^b^
AA	48 (30.5)	30 (24.2)		0.59 (0.35–1.01)
CA	63 (40.1)	62 (50)		
CC	46 (29.2)	32 (25.8)		
Allele C	0.5	0.5		
Allele A	0.5	0.5		
MIR26A-1 (rs7372209)	136	145	0.7627	CC vs. Others ^b^
CC	64 (47.1)	70 (48.3)		0.92 (0.55–1.55)
CT	56 (41.1)	56 (38.6)		
TT	16 (11.8)	19 (13.1)		
Allele C	0.7	0.7		
Allele T	0.3	0.3		

^a^ Bonferroni correction = Plimit: 0.003125; ^b^ The best genetic model according to AIC; * Logistic regression adjusted for confounders: age and sex.

## Data Availability

All relevant data will be shared as Supporting Information files if the manuscript is accepted for publication.

## Supplementary Materials

The following supporting information can be downloaded at: https://www.mdpi.com/article/10.3390/genes14020453/s1, Table S1: Characterization of the 26 variants genetics.
