# Supplementary material for: Impact of pri-let-7a-1 rs10739971 for Gastric Cancer Predisposition in an Amazon Region
Source: Genes (Basel). 2023 Feb 9;14(2):453. doi: 10.3390/genes14020453 (PMC9956087; doi:10.3390/genes14020453)
Supplement: Supplementary file 1 [file genes-14-00453-s001.zip › genes-2025069-supplementary.pdf]

Table S1. Characterization of the 26 variants genetics.

GENE	SNP	ALLELE	LOCATION	QUALITY CONTROL		
				MAF	HWE	GENOTYPE
**AGO1**	rs636832	A > G	Intron variant	36%	0.8508	88%
**DROSHA**	rs639174	T > C	Intron variant	45%	0.00146	87%
**DROSHA**	rs10035440	T > C	Intron variant	15%	0.78762	91%
**DROSHA**	rs3805500	G > A	Intron variant	49%	0	94%
**MIR605**	rs2043556	C > T	Non-coding transcript exon variant	25%	0.00338	84%
**MIR100**	rs1834306	A > G	Intergenic variant	45%	0.71676	87%
**MIR146A**	rs2910164	G > C	Mature miRNA variant	29%	0.86116	93%
**MIR149**	rs2292832	C > T	Non-coding transcript exon variant	38%	0	87%
**MIR196A2**	rs11614913	C > T	Mature miRNA variant	33%	1	86%
**MIR-200B**	rs9660710	C > A	Regulatory region variant	17%	0	89%
**MIR-200C**	rs12904	G > A	3 prime UTR variant	48%	0.33903	85%
**MIR2053**	rs10505168	C > T	Non-coding transcript exon variant	38%	0.10883	80%
**MIR219-1**	rs213210	A > G	Regulatory region variant	17%	0	95%
**MIR219-1**	rs107822	C > T	TF binding site	37%	0.21006	86%
**MIR26A-1**	rs7372209	C > T	Intron variant	20%	0.1847	87%
**MIR300**	rs12894467	T > C	Non-coding transcript exon variant	39%	1	97%
**MIR423**	rs6505162	C > A	5 prime UTR variant	49%	1	89%
**MIR4513**	rs2168518	G>A	Mature miRNA variant	24%	0.07447	94%
**MIR323B**	rs56103835	T > C	Non-coding transcript exon variant	29%	0.81738	87%
**MIR499**	rs3746444	A > G	Mature miRNA variant	18%	0.20179	95%
**MIR570**	rs4143815	G > C	3 prime UTR variant	28%	0.00805	95%
**MIR604**	rs2368392	G > A	Non-coding transcript exon variant	32%	0.00232	76%
**MIR608**	rs4919510	G > C	Mature miRNA variant	36%	0.12564	93%
**PRE-MIR-938**	rs2505901	C > T	Intron variant	40%	0	90%
**PRI-LET-7A-1**	rs10739971	G > A	Intron variant	25%	0.11509	93%
**miR-20b / miR-17-5p (KRT81)**	rs3660	C > G	3 prime UTR variant	20%	0.02217	88%
